# Clinical Evaluation of Acupuncture as Treatment for Complications of Cerebrovascular Accidents: A Randomized, Sham-Controlled, Subject- and Assessor-Blind Trial

**DOI:** 10.1155/2017/7498763

**Published:** 2017-03-20

**Authors:** Hsien-Yin Liao, Wen-Chao Ho, Chun-Chung Chen, Jaung-Geng Lin, Chia-chi Chang, Liang-Yu Chen, De-Chih Lee, Yu-Chen Lee

**Affiliations:** ^1^College of Chinese Medicine, Graduate Institute of Acupuncture Science, China Medical University, Taichung 404, Taiwan; ^2^Department of Acupuncture, China Medical University Hospital, Taichung 404, Taiwan; ^3^Department of Public Health, China Medical University, Taichung 404, Taiwan; ^4^College of Chinese Medicine, China Medical University, Taichung 404, Taiwan; ^5^Department of Neurosurgery, China Medical University Hospital, Taichung 404, Taiwan; ^6^Department of Information Management, Da-Yeh University, Changhua 500, Taiwan; ^7^Research Center for Chinese Medicine & Acupuncture, China Medical University, Taichung 404, Taiwan

## Abstract

*Background and Purpose*. The effect of acupuncture as treatment for poststroke complications is questionable. We performed a randomized, sham-controlled double-blind study to investigate it.* Methods*. Patients with first-time acute stroke were randomized to receive 24 sessions of either real or sham acupuncture during an eight-week period. The primary outcome measure was change in National Institute of Health Stroke Scale (NIHSS) score. Secondary outcome measures included changes in Barthel Index (BI), Instrumental Activities of Daily Living (IADL), Hamilton Depression Rating Scale (HAM-D), and Visual Analogue Scale (VAS) for pain scores.* Results*. Of the 52 patients who were randomized to receive acupuncture (*n* = 28) or placebo (*n* = 24), 10 patients in the acupuncture group and 9 patients in the placebo group failed to complete the treatment. In total, 18 patients in the acupuncture group and 15 patients in the control group completed the treatment course. Reduction in pain was significantly greater in the acupuncture group than in the control group (*p* value = 0.04). There were no significant differences in the other measures between the two groups.* Conclusions*. Acupuncture provided more effective poststroke pain relief than sham acupuncture treatment. However, acupuncture had no better effect on neurological, functional, and psychological improvement.

## 1. Introduction

Stroke is one of the most common causes of death and the leading cause of biopsychosocial disability worldwide. Poststroke complications such as death, loss of motor function, daily life dysfunction, depression, and shoulder pain are common [1]. Although intra-arterial thrombolysis [2], intravenous tissue plasminogen activator [3, 4], and other advanced therapies [5] can reduce the incidence of serious postcomplications when performed within the first few hours of symptom onset, no single therapy has been shown to prevent all complications.

Acupuncture is commonly used in Asian countries to manage poststroke complications, and numerous studies on its effectiveness have been conducted. However, the efficacy of acupuncture as treatment for stroke-related complications has been questioned because only a limited number of well-designed clinical trials have been published. For example, in a recent meta-analysis of eight randomized controlled trials, Park et al. found that the methodological quality of those trials was inadequate to determine the efficacy of acupuncture as treatment for spasticity after stroke [6]. Other meta-analyses have concluded that acupuncture has no beneficial effect on poststroke paralysis [7, 8]. However, some analyses have demonstrated that acupuncture can improve cognitive function and decrease depressive symptoms after stroke [9, 10].

Few studies have employed well-designed protocol or well-established scales to qualitatively measure the effect of acupuncture on biopsychosocial complications after stroke. We, therefore, designed a subject- and assessor-blinded, randomized sham-controlled clinical trial to assess whether acupuncture is an efficacious treatment modality for poststroke neurologic symptoms using the National Institute of Health Stroke Scale, activities of daily living using the Barthel Index and Instrumental Activities of Daily Living Scale, depressive symptoms using the Hamilton Depression Rating scale, and pain severity using the Visual Analogue Scale in patients with recent first-time stroke.

## 2. Methods

### 2.1. Ethics

The study was conducted in accordance with the Declaration of Helsinki. The study was approved by the Research Ethics Committee of the China Medical University Hospital, Taichung, Taiwan (Protocol ID: CMUH102-REC2-015). The study was also registered on ClinicalTrials.gov before commencement (NCT02197663).

### 2.2. Study Design

**Figure 1:**
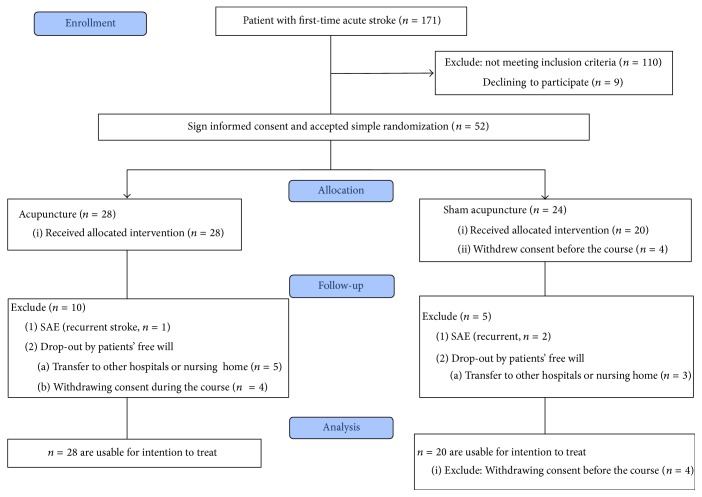
Flow diagram of patient screening in this study.

This was a randomized, sham-controlled, subject- and assessor-blind, single-center exploratory clinical trial to investigate the efficacy of 24 sessions of acupuncture as treatment for complications associated with cerebrovascular accidents. A flowchart outlining patient selection and randomization procedures is shown in Figure 1.

### 2.3. Participants

We had an interview with new stroke patients. During these talks, we checked their response. If patients were unable to cooperate or follow directions for the assessments and tests of our study, they were excluded. Other patients who met the inclusion criteria were given full details of the study in both oral and written form. If the patients agreed to join our study, they needed to sign the informed consent before the beginning of the study. If they were handicapped with limbs weakness, the procedure of signature could be helped by their family.

All patients with first-time incident stroke who were admitted to the Neurological, Neurosurgical, Physical Medicine and Rehabilitation, or Chinese Medicine Departments at the China Medical University Hospital were considered eligible for recruitment. A diagnosis of first-time and recent (1~2 weeks) stroke was based on clinical findings and definitive brain computed tomographic (CT) or magnetic resonance (MR) imaging evidence of ischemic or hemorrhagic stroke. Patients were excluded from participating in the study if they had a history of previous stroke or a history of other serious diseases such as cancer, dementia, heart failure, chronic obstructive pulmonary disease, liver cirrhosis, or kidney failure.

After western physicians confirmed that the vital signs of new stroke patients were relatively stable, both randomization and first-time intervention were conducted within one week after stroke was diagnosed.

### 2.4. Sample Size

The sample size was determined based on an effect size of *d* = 0.833 to detect significance of the intervention effect on the primary outcome measure, namely, change in NIHSS score, as described previously [11]. If we permitted a 5% chance of type I error (*α* = 0.05), a 20% chance of type II error (*β* = 0.2), and a 20% drop-out rate, then approximately 30 initial participants in each group would be required to have a sufficient sample size. All calculations were performed with G^*∗*^power for Windows (Version 3.1, Free Software, Düsseldorf, Germany).

### 2.5. Randomization

Patients were allocated to receive real acupuncture or sham acupuncture following the principle of simple randomization. Lots with numbers 1~60 were placed into a sealed envelope and participants were asked to draw one without looking at the number. Patients who drew an even number were allocated to the treatment group and those who drew an odd number were allocated to the sham acupuncture group. Only one investigator who was not involved in the treatment, assessment, or data analysis could see the number on the lots. The patients, the assessor, and biostatistician were blinded to the allocation until the data were analyzed.

### 2.6. Intervention Group (Acupuncture)

A single practitioner of Chinese Medicine with more than 15 years of experience in acupuncture performed all of the interventions. Manual acupuncture was carried out in patients in the supine position and comprised both body and scalp acupuncture for a total of 20 minutes per session 3 times per week for a total of 24 sessions. The following acupoints were needled in all patients: Baihui (GV-20), Sishencong (EX-HN1), temporal three-needle technique (Jin three-needle therapy, one side for the weakness limbs), Quchi (LI11), Hegu (LI4), Neiguan (PC6), Waiguan (TE5), Yanglingquan (GB34), Zusanli (ST36), Sanyinjiao (SP6), and Taichong (LR3). Other acupoints were needled based on each patient's symptoms, such as Speech II or Speech III areas (Jiao's Scalp Acupuncture) for aphasia, Jinjin (EX-HN 12) and Yuye (EX-HN 13) for dysarthria, and Fenglung (ST40) and Chizexue (LU5) for sputum. Each acupoint was stimulated to elicit a needle sensation (de qi) and needling depth was based on the excitation of de qi. All procedures were carried out with disposable needles measuring 0.25 mm in diameter (32-gauge) and 44 mm in length (Yu Kuang, Taipei, Taiwan).

### 2.7. Control Group (Sham Acupuncture)

Participants in the sham group also received 24 sessions of acupuncture treatment; however, needling was performed 1 cm away from the real acupoints. In addition, none of the participants in the sham group received scalp acupuncture. All procedures were carried out to a depth of 0.5 cm with disposable needles measuring 0.16 mm in diameter (40-gauge) and 12.7 mm in length (Yu Kuang, Taipei, Taiwan). No needle sensation (de qi) was elicited.

Patients in both groups also received conventional western rehabilitation with the same frequency and received western medications as needed during inpatient admission and outpatient tracking.

### 2.8. Outcome Measures

Assessments were carried out before treatment (baseline) and at the end of the 8th, 16th, and 24th treatment session by a single trained, certified, and blinded resident physician.

### 2.9. Primary Outcome Measure

The primary outcome was change in the National Institute of Health Stroke Scale (NIHSS), a well-established measure of stroke severity.

### 2.10. Secondary

Secondary outcome measures included changes in Barthel Index (BI, Mahoney and Barthel Version), Instrumental Activities of Daily Living (IADL, Lawton, M. P., & Brody, E. Version), Hamilton Depression Rating Scale (HAM-D, 24-item Version), and Visual Analogue Scale for pain (VAS) scores. We used the VAS to assess the severity of pain caused by muscle spasticity, limbs hemiplegia, and hemiparesis. Severity of central poststroke pain such as burning, throbbing, or shooting pain was not assessed.

### 2.11. Incidence of Severe Adverse Events

The majority of potential acupuncture-related adverse events are not life-threatening and have documented low incidence rates such as local bleeding (<1%), haematoma (<3%), dizziness (<1%), fainting (<1%), and pain (<10%) [11, 12]. Therefore, in this study, only major adverse events such as recurrent stroke were included in the analysis.

### 2.12. Statistical Analysis

Baseline characteristics are expressed as mean ± standard deviation for continuous variables or as percentages for categorical variables. The chi-square test was used for categorical comparisons of data. Between-group differences in primary and secondary outcome measures are expressed as mean ± standard deviation and were tested by the Mann–Whitney *U* test. For within-group comparisons, the Wilcoxon signed-rank test was used. A *p* value < 0.05 was considered to indicate statistical significance. All statistical analyses were carried out using the statistical package SPSS for Windows (Version 21.0, SPSS, Chicago, Illinois, USA).

The analyses were performed on an intention-to-treat basis, with missing data replaced by the principle of last observation carried forward.

## 3. Results

### 3.1. Recruitment and Severe Adverse Events

Figure 1 summarizes the patient selection, recruitment, and exclusion protocols of the trial. Of the 171 patients with first-time stroke who presented to the CMUH during the period June 2014 to October 2015, 61 met the inclusion criteria and 52 of them provided signed informed consent to participate. The 52 patients were randomly assigned to receive real acupuncture (*n* = 28) or sham acupuncture (*n* = 24). However, after allocation, 4 patients in the placebo group withdrew consent to participate and were, therefore, excluded from the analysis. During the 8-week treatment period, 3 patients were withdrawn from the trial because of recurrent stroke (1 in the acupuncture group and 2 in the sham group) and 12 dropped out for personal reasons (9 in the acupuncture group and 3 in the sham group), including transfer to other institutions (*n* = 5 and *n* = 3, resp.) and withdrawal of consent (4 in the acupuncture group). Therefore, 18 patients in the acupuncture group and 15 patients in the sham group completed the treatment course. However, for intention-to-treat purposes, data on 28 participants in the acupuncture group and data on 20 participants in the sham group were available for analysis.

### 3.2. Baseline Data

Baseline data are presented in Table 1. There were no significant differences in baseline characteristics between the two groups of patients with the exception of stroke type. The incidence of ischemic stroke was significantly higher in the sham group than in the acupuncture group and the incidence of hemorrhagic stroke was significantly higher in the acupuncture group than in the sham group.

### 3.3. Drop-Out Rate and Drop-Out Data Analysis

As seen in Figure 1, the drop-out rates were 32.14% (9/28) in the acupuncture group and 29.17% (7/24) in the sham group. No one dropped out due to the design or execution of the trial.

### 3.4. Primary and Secondary Outcome Measures

By analyzing the data after 8th and 16th treatment sessions, we found no significant between-group differences and decided to present the data of baseline and the end.

**Figure 2:**
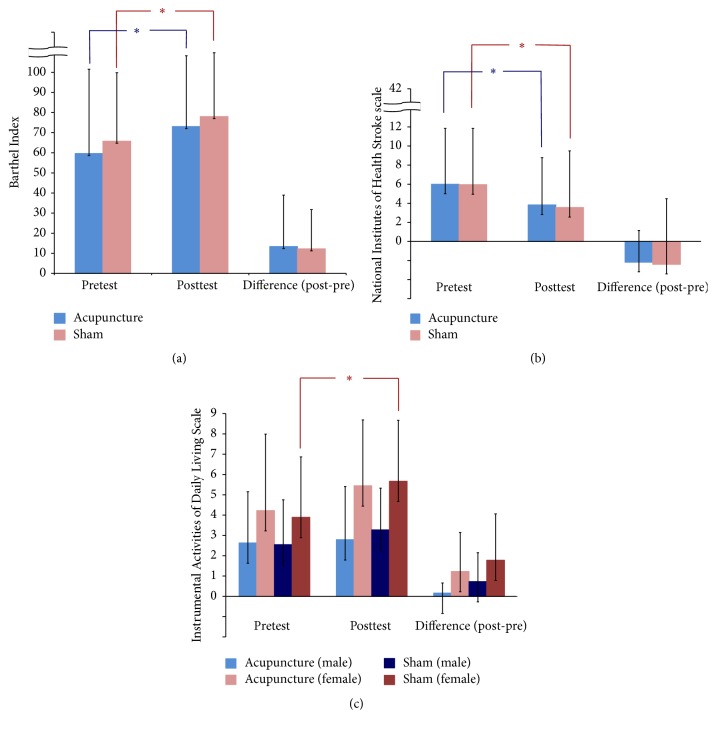
(a) Effect of acupuncture and sham acupuncture on BI, ^*∗*^*p* < 0.05. (b) Effect of acupuncture and sham acupuncture on NIHSS, ^*∗*^*p* < 0.05. (c) Effect of acupuncture and sham acupuncture on IADL, ^*∗*^*p* < 0.05.

**Figure 3:**
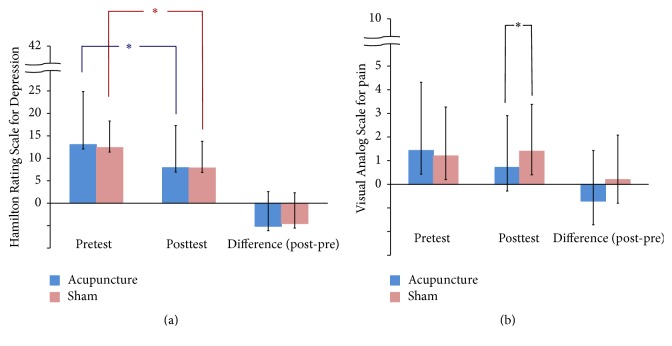
(a) Effect of acupuncture and sham acupuncture on HAM-D, ^*∗*^*p* < 0.05. (b) Effect of acupuncture and sham acupuncture on VAS, ^*∗*^*p* < 0.05.

As shown in Figures 2(a), 2(b), and 3(a), intragroup comparisons revealed that both acupuncture group and sham acupuncture group had significant improvements in NIHSS, Barthel Index, and HAM-D scores. However, there were no significant differences in improvement in scores between the two groups.

As shown in Figure 2(c), there were no significant intergroup or intragroup differences in scores on the IADL scale. Interestingly, however, significant intragroup improvement in IADL scale was noted for women who received sham acupuncture.

As shown in Figure 3(b), reduction in pain was significantly greater in the acupuncture group than in the control group (*p* = 0.04).

Whole results of all scales were shown as Table 2.

## 4. Discussion

In this study, we found no significant differences in change in NIHSS, BI, IADL, or HAM-D scores between patients who received real acupuncture and those who received sham treatment. The high drop-out rate is one of the possible causes for that finding. The attrition rate was estimated to be 20%; however, the rates were 28.57% in the acupuncture group and 29.17% in the sham group. One of the reasons for the higher than expected attrition rates was the relatively high number of acupuncture sessions (*n* = 24) required to complete the trial. Patients who were transferred to other hospitals for western treatment during the trial might have found it inconvenient to return to the CMUH to receive our treatment due to either distance or time constraints and, therefore, dropped out of the study. Liu et al. found that acupuncture did not have a beneficial effect on stroke recovery and that the lack of effect might have been because the assessments of the effect were conducted too soon after the occurrence of stroke, in most cases 2-3 weeks after stroke [13]. That was the reason why we designed the trial to include 24 treatment sessions. Another possible reason for the lack of better effect of acupuncture on the majority of poststroke complications is that patients in both groups underwent conventional western rehabilitation and were given western medications as needed during inpatient admission and outpatient tracking, which may have introduced bias. In addition, the effect of western treatment might have masked or minimized the between-group differences in all scales. A third reason is due, at least in part, to our limited research resources; we were unable to recruit enough patients to achieve the minimal sample size needed to detect significance of the intervention effect on the primary outcome measure, namely, change in NIHSS.

Two types of pain are common poststroke complications. One is central poststroke pain and the other is hemiplegic shoulder pain, which impacts the limb motor function, disturbs the rehabilitation process, extends the length of hospitalization, and affects the patient's quality of life. Because we excluded central poststroke pain at first, lots of the pain as our study showed are hemiplegic shoulder pain and fewer are pain from affected lower limbs. Previous study has shown that spastic symptoms can induce pain, ankylosis, tendon retraction, or muscle weakness [14]. Besides, hemiplegic shoulder pain affects 16–84% of impaired stroke survivors [15]. Even though there are lots of therapies such as physiotherapy, massage, strapping, slings, intraarticular or subacromial corticosteroid injections, suprascapular nerve blocks, percutaneous or superficial electrical muscle stimulation, and intramuscular botulinum toxin type A injections, there is no specific evidence for most of these treatments [16]. Our trial focused on the pain caused by spasticity and showed that acupuncture is effective at reducing the severity of limb pain. Two recent systematic reviews also concluded that acupuncture is an effective treatment modality for poststroke shoulder pain [17] and spasticity [18]. The obvious between-group difference in posttest VAS scales might indirectly show that acupuncture results in a reduction in the degree of pain caused by spasticity.

Furthermore, to highlight the value of our study, we searched for clinical trials of acupuncture for global functional recovery from acute to subacute stage of stroke conducted during the period 1992 to 2016 and identified nine studies with relatively good methodological quality.  Table 3 provides a summary and comparison of these trials. Among those nine trials, three were adequately designed subject-and-assessor-blind studies with a low degree of performance bias [11, 19, 20]. In our subject-and-assessor-blind trial, we minimized potential bias by excluding patients with recurrent stroke, unlike the trials conducted by Naeser et al., Gosman-Hedström et al., Johansson et al., and Schuler et al. Furthermore, our use of only one therapist and one blind assessor further minimized treatment and detection bias. The number of acupuncture sessions in our study (*n* = 24) conducted over an eight-week period was markedly higher than in most other trials, allowing us to more accurately measure the accumulation effect of acupuncture on stroke-related complications. As in most of the previous trials on the effect of acupuncture in stroke patients, our trial used intention-to-treat to avoid attrition bias when analyzing data. In addition, as in most trials, we found that acupuncture resulted in modest improvement in some of the outcome measures, such as the Visual Analogue Scale for pain.

## 5. Conclusion

Acupuncture provided more effective poststroke pain relief than sham acupuncture treatment in the subacute phase of stroke. However, acupuncture was not superior to sham treatment for recovery of neurological function, activities of daily living, or depression. Further studies involving larger sample sizes and more treatment sessions are warranted.

## Figures and Tables

**Table 1:** Baseline characteristics of randomized subjects.

	Acupuncture (*n* = 28)	Sham acupuncture (*n* = 20)	*p* value
Male, *n* (%)	19 (67.86%)	11 (55.00%)	0.385
Female, *n* (%)	9 (33.33%)	9 (45.00%)	
Image finding: infarct, *n* (%)	14 (50%)	17 (80%)	0.016^*∗*^
Image finding: hemorrhage, *n* (%)	14 (50%)	3 (20%)	
Hypertension, *n* (%)	22 (78.57%)	13 (65.00%)	0.34
Diabetes, *n* (%)	5 (17.86%)	6 (30.00%)	0.49
Heart disease, *n* (%)	5 (17.86%)	4 (13.30%)	1.00
Hyperlipidemia, *n* (%)	0 (0.00%)	1 (5.00%)	0.42
Age (mean ± SD)	62.29 ± 12.33	55.45 ± 15.22	0.09
Height (mean ± SD)	162.36 ± 7.58	163.03 ± 8.50	0.78
Weight (mean ± SD)	64.56 ± 9.67	68.84 ± 11.95	0.18
Prescore of Barthel Index (mean ± SD)	59.64 ± 41.94	65.75 ± 34.08	0.93
Prescore of Instrumental Activities of Daily Living (male) (mean ± SD)	2.63 ± 2.52	2.55 ± 2.21	1.00
Prescore of Instrumental Activities of Daily Living (female) (mean ± SD)	3.8 ± 3.5	3.4 ± 2.5	1.00
Prescore of National Institutes of Health Stroke Scale (mean ± SD)	6.00 ± 5.84	6.93 ± 5.75	0.22
Prescore of Hamilton Depression Rating Scale (mean ± SD)	13.94 ± 11.27	12.6 ± 6.34	0.62
Prescore of Visual Analogue Scale for pain (mean ± SD)	1.56 ± 2.97	1.47 ± 2.29	0.68

**Table 2:** Comparison of NIHSS, Barthel Index, IADL, HAM-D, and VAS scores between two groups.

Group assessment	Acupuncture		Sham		*p* value (between-group)	
	Pretest	Posttest	Pretest	Posttest	Pretest	Posttest
NIHSS	6.00 ± 5.84	3.82 ± 4.95	5.95 ± 5.45	3.55 ± 4.63	0.67	0.97
NIHSS difference	−2.18 ± 3.32		−2.40 ± 2.62		0.59	

Barthel Index	59.64 ± 41.94	73.04 ± 35.23	65.75 ± 34.08	78.00 ± 31.72	0.93	0.60
BI difference	13.39 ± 25.57		12.25 ± 19.50		0.40	

IADL (male)	2.63 ± 2.52	2.79 ± 2.62	2.55 ± 2.21	3.27 ± 2.05	1.00	0.70
IADL (male) difference	0.16 ± 0.50		0.73 ± 1.12		0.42	

IADL (female)	4.22 ± 3.77	5.44 ± 3.25	3.89 ± 2.98	5.67 ± 3.00	1.00	0.93
IADL (female) difference	1.22 ± 1.92		1.78 ± 2.279		0.73	

HAM-D	13.94 ± 11.27	6.22 ± 6.61	12.60 ± 6.34	6.53 ± 5.80	0.62	0.50
HAM-D difference	−7.72 ± 8.37		−6.07 ± 7.11		0.97	

VAS	1.56 ± 2.97	0.44 ± 1.83	1.47 ± 2.29	1.73 ± 2.15	0.68	0.04^*∗*^
VAS difference	−1.11 ± 2.54		0.27 ± 2.11		0.53	

Wilcoxon signed rank test was used. ^*∗*^*p* < 0.05.

NIHSS: National Institutes of Health Stroke Scale; BI: Barthel Index; IADL: Instrumental Activities of Daily Living; HAM-D: Hamilton Depression Rating Scale; VAS: Visual Analogue Scale for pain.

**Table 3:** Summary and comparison of randomized sham-controlled trials of acupuncture for global functional recovery after stroke (acute to subacute stage).

Study	Study design	Patient population	Experimental treatment	Control treatment	Therapist/session of course	Assessor/ITT or PP/outcome measures	Intergroup differences
Naeser et al. 1992 [19]	Double-blind, parallel 2-arm	*n* = 16, 1–3 mth after infarction Fresh case?	(A) (*n* = 10) Acupuncture + electroacupuncture and scalp acupuncture	(B) (*n* = 6) Sham acupuncture (penetrating) + sham electrical stimulation (electrical stimulation not conducted)	Therapists? 20 times Within 4 wks	3 assessors not belonging to ITT or PP? (1) Boston Motor Inventory (2) Boston Motor Inventory with analysis of CT scan of lesion site	Significantly dependent on CT scan lesion site

Gosman-Hedström et al. 1998 [21]	Single-blind, parallel 3-arm	*n* = 104, <1 wk after stroke Fresh case?	(A) (*n* = 37) (electrical + manual) Acupuncture + conventional stroke rehabilitation	(B) (*n* = 34) Superficial acupuncture, no “de chi” + conventional stroke rehabilitation (C) (*n* = 33) Conventional stroke rehabilitation	4 therapists 20 times Within 10 wks	2 assessor intention to treat (1) Scandinavian Neurological Stroke Scale (2) Barthel ADL Index (3) Sunnaas ADL Index (4) Nottingham Health Profile	Not significant

Johansson et al. 2001 [22]	Single-blind, parallel 3-arm	*n* = 150 5~10 d after stroke Fresh case?	(A) (*n* = 48) Acupuncture + electroacupuncture + conventional physiotherapy (B) (*n* = 51) High-intensity, low-frequency TENS + conventional physiotherapy	(C) (*n* = 51) Low-intensity, high-frequency TENS + conventional physiotherapy	Therapists from 7 university and district hospitals 20 times Within 10 wks	1 assessor intention to treat (1) Barthel Index (2) Rivermead Mobility Index (3) Nine-Hole Peg Test (4) Nottingham Health Profile	Not significant

Park et al. 2005 [20]	Double-blind, parallel 2-arm	*n* = 116, <4 wk after stroke Fresh case	(A) (*n* = 56) Acupuncture	(B) (*n* = 60) Sham acupuncture (nonpenetrating on nonacupuncture points)	1 therapist 9–12 times Within 2 wks	4 assessors intention to treat (1) Barthel ADL Index (2) NIHSS (3) Motoricity Index (4) EQ-5D (5) Nottingham Extended Activities of Daily Living (6) Modified Ashworth Scale	Not significant Except a greater improvement in leg function

Schuler et al. 2005 [23]	Single-blind, parallel 3-arm	*n* = 120, 3–35 d after stroke Fresh case?	(A) (*n* = 41) Acupuncture + electrical stimulation	(B) (*n* = 40) Sham electroacupuncture (surface electrodes on acupuncture points with visual stimulation) (C) (*n* = 39) Control (no additional treatment)	3 therapists 8 times Within 4 wks	Assessors? Intention to treat (1) European Stroke Scale (2) Barthel Index	No differences

Hopwood et al. 2008 [24]	Single-blind, parallel 2-arm	*n* = 105, 4–10 d after stroke Fresh case	(A) (*n* = 57) Acupuncture + electroacupuncture and scalp acupuncture	(B) (*n* = 48) Sham TENS (with no current flow)	therapists from 5 general hospitals in Hampshire (UK) 12 times Within 4 wks	assessment nurses intention to treat (1) Barthel Index (2) Motoricity Index (3) Nottingham Health Profile	no significant difference Except improvement in the MI

Shen et al. 2012 [11]	Double-blind, parallel 2-arm	*n* = 290, 24 hr–14 d after acute ischemic stroke Fresh case	(A) (*n* = 145) Acupuncture + medical treatment	(B) (*n* = 145) Sham acupuncture (nearby the acupoints) + medical treatment	Therapists of 4 hospitals 28 times Within 4 wk	A research nurse ITT or PP? (1) Barthel Index (2) Relapse (3) Death (4) NIHSS (5) Chinese Stroke Scale (6) Oxford Handicap Scale (7) Stroke Specific Quality of Life Scale	(1) *p* = 0.01 (2) *p* = 0.001 (3) *p* = 0.558 (4) *p* < 0.01 (5) *p* < 0.001 (6) Intervention < control (7) *p* < 0.01

Zhu et al. 2013 [25]	Single-blind, parallel 2-arm	*n* = 188 <30 d after stroke Fresh case	(A) (*n* = 98) Acupuncture (body and scalp) + conventional physiotherapy	(B) (*n* = 90) Conventional physiotherapy	4 acupuncturists and 4 physiatrists? From 4 rehabilitation centers 42.6 times of body acupuncture and 22.5 times of scalp acupuncture within 3 mths	4 physiatrists Intention to treat (1) Fugl-Meyer Assessment (2) Barthel Index	No significant difference

Zhang et al. 2015 [12]	Single-blinded, parallel 2-arm	*n* = 862 3–10 d after ischemic stroke Fresh case?	(A) (*n* = 427) Acupuncture plus standard care	(B) (*n* = 435) Standard care	Acupuncturists of multicenter (40 hospitals) 20 times Within 4 wks	Assessors? Intention to treat (1) Scandinavian Stroke Scale (2) Barthel Index	No significant difference

Our study	Double-blind, parallel 2-arm	*n* = 52, 1~2 wk After stroke Fresh case	(A) (*n* = 28) Acupuncture (body and scalp) + conventional physiotherapy	(B) (*n* = 24) Sham acupuncture (superficial, not acupoints and no “de chi”) + conventional physiotherapy	1 therapist, 24 times Within 8 wks	1 assessor Intention to treat (1) NIHSS (2) Barthel Index (3) Instrumental Activities of Daily Living (4) Hamilton Rating Scale for Depression (5) Visual Analogue Scale for pain	(1) *p* = 0.97 (2) *p* = 0.60 (3) *p* = 0.70 male *p* = 0.93 female (4) *p* = 0.50 (5) *p* = 0.04

TENS: transcutaneous electrical nerve stimulation; NIHSS: National Institutes of Health Stroke Scale; d: day; wk: week; mth: month.

World Health Organization Quality of Life BREF (WHOQOL-BREF); ITT: intention to treat; PP: per-protocol.

## Abbreviations

NIHSS: National Institutes of Health Stroke Scale
BI: Barthel Index
IADL: Instrumental Activities of Daily Living
HAM-D: Hamilton Depression Rating Scale
VAS: Visual Analogue Scale for pain
SAE: Severe Adverse Events
CMUH: China Medical University Hospital.
